# Comprehensive smartphone image dataset for fish species identification in Bangladesh's freshwater ecosystems

**DOI:** 10.1016/j.dib.2025.111629

**Published:** 2025-05-12

**Authors:** Saieef Sunny, Shiam Prodhan, Nazmuj Shakib, Mohammad Rifat Ahmmad Rashid, Nafees Mansoor

**Affiliations:** a Department of Computer Science and Engineering, University of Liberal Arts Bangladesh, Mohammadpur, Dhaka, Bangladesh; b Department of Computer Science and Engineering, East West University, Aftabnagar, Dhaka, Bangladesh

**Keywords:** Fish species identification, Dataset, Freshwater fish, Bangladesh, Deep learning, Machine learning, Image recognition, Biodiversity

## Abstract

The Bangladesh Fish Species Identification dataset encompasses 24,925 images representing 21 freshwater species commonly found in the rivers and ponds of Bangladesh. Photographs were taken using smartphones under carefully controlled conditions to ensure clarity and proper framing, and they include variations in lighting and background for realistic depiction. Species covered in this dataset include Aair, Boal, Chapila, DeshiPuti, Foli, Ilish, KalBaush, Katla, Koi, Magur, Mrigel, Pabda, Pangas, Puti, Rui, Shol, Shorputi, Taki, Tarabaim, Telapiya, and Tengra. By providing an extensive collection of labeled, high-quality images, this dataset serves as a valuable foundation for research on aquatic biodiversity, fishery management, and the development of precise machine-learning models for species recognition. It supports investigations into morphological variations, facilitates the evaluation of deep learning techniques, and assists in understanding fish distribution across diverse habitats.

Specifications Table

**Table:** 

Subject	Dataset of images for fish species recognition in Bangladesh
Specific subject area	Fish species identification using deep learning techniques
Data format	Raw
Type of data	Image
Data collection	This dataset comprises 24,925 high-resolution images of 21 commonly encountered freshwater fish species in Bangladesh. Images were collected in local markets, primarily at Shoari Ghat in Dhaka and Nagaon Bazar in the Naogaon district, using a smartphone camera to capture each species from multiple angles and under varying lighting conditions. All images were uniformly resized to 600 × 600 pixels for storage and processing, saved in JPEG format, and renamed in a sequential manner to ensure clarity and consistency. No specialized hardware or software tools were used, and no normalization techniques were applied, allowing the dataset to retain the authenticity and variability present in the original photographs.
Data source location	Shoari Ghat Fish Market, Dhaka, Bangladesh (Latitude: 23° 42′ 42.0198″, Longitude: 90° 23′ 41.683′') Nagaon Bazar Fish Market, Naogaon, Bangladesh (Latitude: 26° 24′ 2.158′', Longitude: 92° 41′ 22.262′')
Data accessibility	Repository name: Mendeley Data Data identification number: doi:10.17632/8r24222wcc.2 Direct URL to data: https://data.mendeley.com/datasets/8r24222wcc/2

## 1 Value of the Data

• The dataset is an important research tool for Bangladeshi fish species identification and biodiversity conservation, facilitating a better understanding of aquatic ecosystems.
• This dataset offers an extensive image dataset for training and assessing machine learning algorithms, enabling the creation of precise and effective fish recognition systems.
• The dataset benefits fisheries management, aquaculture, and environmental science researchers by enabling data-driven decision-making and policy formulation [1].
• Using the dataset, biologists, computer scientists, and conservationists can collaborate interdisciplinary to address urgent problems in aquatic resource management.
• It advances the state-of-the-art in automated species identification by facilitating benchmarking studies and comparative analyses across various fish species recognition techniques [2].

## 2 Background

Bangladesh, a nation traversed by numerous rivers, maintains a longstanding cultural connection with fish, reflected in the phrase “Mache Bhate Bangali” [3]. Fish is a primary component of the national diet and serves as a significant source of nutrition [4]. Nevertheless, many individuals, especially those from younger demographics, lack adequate understanding of common species such as Rui, Katla, and Ilish [5]. The country’s extensive aquatic biodiversity—encompassing both freshwater and saltwater varieties due to broad river networks and the presence of one of the world’s largest seaports—emphasizes the need for enhanced fish identification resources [6]. Insufficient awareness of local freshwater species often leads to uncertainty among younger buyers and discourages consumption, driven by concerns over misinformation. As dependence on technology grows, a user-friendly fish identification platform represents a practical avenue for bridging this knowledge gap, reinforcing consumer confidence, encouraging responsible selling practices, and preserving the cultural and nutritional significance of fish in Bangladesh. Technology holds considerable potential in ensuring that consumers remain well-informed and assured when selecting fish. Table 1 provides an overview of existing fish image datasets related to Bangladesh. The Proposed Dataset, titled “Bangladeshi Fish Species Identification Dataset," spans 24,925 images of 21 freshwater species.

**Table 1:** Papers on fish image datasets and studies in Bangladesh.

No.	Paper Id	Dataset	Images (Instances) Size	Categories	Aim of Dataset
1	[11]	BDFreshFish	3100 images (300 augmented/class)	8 native freshwater species	To support deep learning and machine learning research on classifying native Bangladeshi freshwater fish, highlighting features like head, body, scales, and fins.
2	[5]	Custom dataset for DL systems	16,000 images	Commonly consumed freshwater species	To identify and classify Bangladeshi freshwater fish using deep learning models for sustainability and fishery management.
3	[12]	BanFish Dataset	2506 images	18 fish species	Aims to facilitate automatic fish classification and preserve cultural knowledge about traditional fish species.
4	Proposed Dataset	Bangladeshi Fish Species Identification Dataset	24,925 images	21 freshwater species	To enable machine learning–based fish identification systems that accurately recognize diverse Bangladeshi freshwater species for informed consumer choices and responsible fishery practices.

## 3 Data Description

This dataset comprises high-resolution images of various fish species commonly found in Bangladesh, as shown in Table 2. The images were gathered from local fish markets, labeled according to species, and organized following the folder structure and sample distribution detailed in Table 3. In total, the dataset contains 24,925 images.

**Table 2:** Sample images and description of each category.

SL.	Category	Scientific Name	Description	Image
1	Aair (Long Whiskered Catfish)	*Sperata aor (Hamilton, 1822)*	The Aair fish, also referred to as the Ayer or Gang major in the local dialect, is a large, scaleless catfish distinguished by its robust body and long whiskers. This species, primarily found in Bangladeshi rivers like the Padma, Meghna, and Jamuna, prefers deeper waters to survive. The fish is found in many places, such as the Sunamganj Haor area, the Halda River in the Chittagong Hill Tracts, and the Chalan Beel in Natore. The aair fish is common in Bangladesh's varied aquatic environments due to its adaptability to various freshwater habitats, from rivers to haors [7].	
2	Boal (Freshwater Shark)	*Wallago attu (Bloch and Schneider, 1801)*	The Boal fish, named for its long body and skin devoid of scales, is sometimes called the freshwater shark because of its large size and predatory habits. The Padma, Meghna, and Jamuna rivers are just a few of the Bangladeshi rivers, haors, and baors where this fish is common. It can grow in a range of freshwater environments, from still pools of water to deep river channels. Occurring all over the country, the Boal is a common sight in places like the Sunamganj Haor area, the Halda River in the Chittagong Hill Tracts, and the Chalan Beel in Natore [7].	
3	Chapila (Indian River Shad)	*Gudusia chapra* (Hamilton, 1822)	The Chapila fish, known locally as Chapila or Khoria, is a small, slender, and scaleless species with a maximum length of around 200 mm. It is native to Bangladesh and is commonly found in rivers like the Padma, Meghna and the haor regions such as Chalan Beel in Natore and Sunamganj. Characterized by its laterally compressed body and silvery appearance, the Chapila thrives in freshwater environments, including rivers and baors. This fish is significant for local fisheries and plays a crucial role in the aquatic ecosystems of Bangladesh [7].	
4	DeshiPuti (Swamp Barb)	*Puntius chola* (Hamilton, 1822)	The little, colorful Chala Punti, Chal Puti, or Puti, distinguished by its silver body and characteristic barbels, is abundant in Bangladesh's freshwater environments. It is frequently observed in areas like the Padma River in Rajshahi, the Chalan Beel in Natore, and the Someshwari River in Netrokona. It can be found in various water bodies, including rivers, beels, and canals. This species favors areas with a lot of flora because it can provide cover and food. The fact that it may be found in a variety of aquatic environments demonstrates how adaptable and important it is ecologically in Bangladesh [7].	
5	Foli (Bronze Featherback)	*notopterus (Pallas, 1769)*	The Foli fish is a distinctive freshwater fish with a sleek, elongated body and a bronze hue. This species is native to the rivers, canals, beels, and haors of Bangladesh, making it a common sight in the Padma, Meghna, and Buriganga rivers, as well as in the Chalan Beel and Halda River. Known for its lateral compression and long anal fin, the Bronze Featherback thrives in slow-moving or stagnant waters, often hiding among submerged vegetation. Its presence across various aquatic environments highlights its adaptability and importance in Bangladesh's diverse aquatic ecosystems [7].	
6	Ilish (Hilsa Shad)	*Tenualosa ilisha (Hamilton, 1822)*	The Ilish, commonly known as Hilsa, is the national fish of Bangladesh. Its elongated body and silvery scales distinguish it. This species can also be found in bay waters and coastal regions; it primarily lives in big rivers and estuaries like the Padma, Meghna, and Jamuna rivers. The ilish is an anadromous fish that migrates north to spawn in freshwater rivers. This species, which does well in both freshwater and brackish conditions, is widely distributed across the rivers and estuaries of Bangladesh. Its life cycle and regional abundance depend heavily on its migratory habits and preferred habitats [7].	
7	KalBaush (Orange Fin Labeo)	*Labeo calbasu (Hamilton, 1822)*	The Kalibaus fish is known for its distinctive dark body and bright orange fins. It inhabits various freshwater bodies throughout Bangladesh, including rivers such as the Padma, Meghna, and Jamuna, haors, beels, and canals. The Kalibaus is particularly prevalent in regions like as Rajshahi, Sunamganj, and the Chittagong Hill Tracts. This fish prefers slow-moving or stagnant waters where it feeds on algae, aquatic plants, and small invertebrates, contributing to the aquatic ecosystem's health [7].	
8	Katla (Catla)	*Labeo catla* (Hamilton, 1822)	The Catla fish, also known locally as Katla or Katal, is a prominent freshwater species in Bangladesh. Recognizable by its broad head and upturned mouth, Catla is often found in rivers, beels, haors, and baors. Common habitats include the Padma River in Rajshahi, the Meghna River in Narsingdi, and the Chalan Beel in Natore. This fish prefers flowing waters and is an essential part of the aquatic biodiversity in regions like the Halda River and the Sunamganj Haor area. Its robust body and distinct appearance make it a significant species in Bangladesh's freshwater ecosystems [8].	
9	Koi (Climbing Perch)	*Anabas testudineus* (Bloch, 1792)	The Koi fish, locally known as Koi or Thai Koi, is a small, robust species characterized by its ability to survive in low-oxygen environments, thanks to its specialized labyrinth organ. Native to Bangladesh, this fish is found in various freshwater habitats, including rivers, canals, and beels. It is particularly common in the Padma River in Rajshahi, the Chalan Beel in Natore, and the Halda River in the Chittagong Hill Tracts. The Koi fish is also noted for its climbing ability, allowing it to traverse short distances over land between water bodies [8].	
10	Magur (Walking Catfish)	*Clarias batrachus* (Linnaeus, 1758 Jan)	The Magur fish, locally known as Mojgur or Jagur, is a species of walking catfish notable for its ability to move overland using its pectoral fins. This hardy fish is scaleless, with a long, cylindrical body and a broad, flat head. Found abundantly in the freshwater rivers, lakes, and floodplains of Bangladesh, Magur thrives in the Padma, Meghna, and Buriganga rivers, among others. Its resilience allows it to inhabit diverse environments, from muddy bottoms of ponds to slow-moving rivers, making it a common sight across various aquatic habitats in the region [8].	
11	Mrigel (Mriga)	*Cirrhinus mrigala* (Hamilton, 1822)	The Mrigel fish, locally known as Mriga or Mrigala, is a prominent freshwater species found in Bangladesh's rivers and lakes. Its silvery scales and sleek physique distinguish this fish. Mrigel is frequently found in rivers, including the Halda, Padma, and Kaptai Lake. It adapts well to the slow-moving waters of rivers and reservoirs and grows in a variety of freshwater settings. The Mrigel makes a substantial contribution to the local fisheries and ecology, making it an essential component of Bangladesh's aquatic biodiversity [8].	
12	Pabda (Butter Catfish)	*Ompok bimaculatus* (Bloch, 1794)	The Pabda fish, or Kani Pabda, is a slender, scaleless species with distinctive spots and elongated whiskers. Found in Bangladesh's freshwater environments, it thrives in rivers such as the Padma, Meghna, and Chitra and haors and beels. Notable for its adaptability, the Kani Pabda is prevalent in diverse habitats, from the Chalan Beel in Natore to the Tanguar Haor in Sunamganj. This fish's widespread presence highlights its ecological versatility and importance in Bangladesh's aquatic biodiversity [8].	
13	Pangas (Yellowtail Catfish)	*pangasius* (Hamilton, 1822)	The Pangas fish, known for its yellow tail, is a significant species in Bangladesh's freshwater ecosystems. This scaleless catfish can grow up to 1 m in length and weigh around 10 kg. Predominantly found in large rivers and estuaries, it thrives in the Padma, Meghna, and Buriganga rivers, among others. Its presence is noted in various regions including Rajshahi, Natore, and Chittagong Hill Tracts. The Pangas fish's adaptability to different aquatic habitats underscores its ecological importance and widespread distribution across Bangladesh [8].	
14	Puti (Spotfin Swamp Barb)	*Puntius sophore* (Hamilton, 1822)	The Spotfin Swamp Barb is a small, colorful fish typically found in freshwater bodies. It is colloquially known as Punti or Jat Punti in Bangladesh. It lives in beels, ponds, and rivers all over the nation, including as the Halda River in the Chittagong Hill Tracts and the Padma River in Rajshahi. A streamlined body and a characteristic patch close to the dorsal fin distinguish this species. The Spotfin Swamp Barb is a vital component of the aquatic ecology in the Sunamganj Haor region and the Kaptai Lake. It is widely spread in these areas [8].	
15	Rui (Rohu)	*Labeo rohita* (Hamilton, 1822)	The Rohu fish, also called Rui in Bangladesh, is a well-known freshwater fish distinguished by its long body and silvery scales. It usually inhabits Bangladesh's rivers, canals, and beels, such as the Padma, Meghna, and Jamuna. This species adapts well to both wild and cultivated situations and thrives in a wide range of freshwater habitats. The Rohu fish is characterized by its enormous size, ability to grow up to 90 cm long, and vital role in regional fisheries and ecosystems [9].	
16	Shol (Striped Snaked)	*Channa striata* (Bloch, 1793)	The Shol fish is a strong, elongated freshwater fish that is widespread in Bangladesh. The Shol, which has a characteristic striped pattern, lives in a variety of water habitats, including as rivers, beels, and canals. It is typically found in the Chalan Beel in Natore, the Padma River in Rajshahi, and the Halda River in the Chittagong Hill Tracts. The Shol is an abundant species in Bangladesh's varied freshwater environments because of its adaptability and resilience, allowing it to flourish in stagnant and moving waterways [9].	
17	Shorputi (Olive Barb)	*Systomus sarana* (Hamilton, 1822)	The Sarputi is a freshwater fish distinguished by its olive-green body and distinctive barbels near the mouth. Predominantly found in rivers, haors, baors, and beels across Bangladesh, this species thrives in clear, slow-moving waters. Significant populations exist in the Padma River, Chalan Beel, and Sunamganj Haor region. The Sarpunti's presence across diverse aquatic habitats highlights its adaptability, making it a familiar sight in the aquatic ecosystems of Bangladesh [9].	
18	Taki (Spotted Snakehead)	*Channa punctata* (Bloch, 1793)	The Tataki, or Taki fish, is distinguished by its elongated body and characteristic dark dots. This fish is indigenous to Bangladesh and can be found in bells, haors, rivers, and other freshwater habitats. The Chalan Beel in Natore, the Halda River in the Chittagong Hill Tracts, and the Padma River in Rajshahi are among the locations where the Taki is frequently found. It is a species adaptable to Bangladesh's varied aquatic environments since it can live in stagnant and moving rivers [9].	
19	Tarabaim (Lesser spiny eel)	*Macrognathus aculeatus* (Bloch, 1786)	The Tarabaim fish, locally known as Golchi or Kota-baim, is a slender, elongated freshwater fish notable for its distinctive pattern and spines along the dorsal fin. It primarily inhabits canals, beels, baors, and rivers throughout Bangladesh, including regions such as the Padma River in Rajshahi, Chalan Beel in Natore, and the Halda River in the Chittagong Hill Tracts. Tara Baim is commonly found in the diverse aquatic environments of the country, thriving in both slow-moving and still waters [9].	
20	Telapiya (Tilapia)	*Oreochromis mossambicus* (Peters, 1852)	The tilapia fish, sometimes referred to as telapiya, is a hardy and versatile species that is frequently found in Bangladesh's many freshwater settings. The three main rivers that contain it are the Ghaghat River in Gaibandha, the Punarbhaba River in Dinajpur, and the Banar River in Mymensingh. This species is well-known for its capacity to adapt to varied water conditions and for thriving in a variety of aquatic settings, including ponds and rivers. The tilapia's strong body and unique laterally compressed shape help it survive in various habitats [9].	
21	Tengra (Mystus catfish)	*Mystus tengara (Hamilton, 1822)*	The Tengra fish, sometimes called Ghuitta-tengra or Tengra locally, is a small, scaleless catfish that may grow up to 12 cm long and is distinguished by its unique striped pattern. It is mostly found in Bangladesh's rivers, haors, and canals and does well in various freshwater environments. The Chalan Beel in Natore, the Padma and Meghna rivers, and several haors in Sunamganj are home to the Tengra fish. Because of its versatility, it can live in various settings, which adds to its extensive prevalence in Bangladesh's aquatic ecosystems [9].

**Table 3:** Overview of sample distribution and folder organization for the dataset.

SL.	Category (Bengali Name)	No. of Original Images	No. of Augmented Images	Folder Name (both for Original and Augmented Images)
1	Aair	1804	10,824	Aair
2	Boal	1651	9906	Boal
3	Chapila	428	2568	Chapila
4	DeshiPuti	412	2472	DeshiPuti
5	Foli	562	3372	Foli
6	Ilish	1031	6186	Ilish
7	KalBaush	917	5502	KalBaush
8	Katla	1765	10,590	Katla
9	Koi	842	5052	Koi
10	Magur	574	3444	Magur
11	Mrigel	1808	10,848	Mrigel
12	Pabda	1764	10,584	Pabda
13	Pangas	934	5604	Pangas
14	Puti	1560	9360	Puti
15	Rui	2726	16,356	Rui
16	Shol	1424	8544	Shol
17	Shorputi	44	264	Shorputi
18	Taki	2223	13,338	Taki
19	Tarabaim	1262	7572	Tarabaim
20	Telapiya	2058	12,348	Telapiya
21	Tengra	1431	8586	Tengra

To ensure high image quality and compatibility across the dataset, all captured images were meticulously saved in JPG format and resized to a standard resolution of 600 × 600 pixels. This uniform approach was implemented to enable seamless integration with a variety of machine-learning applications, thereby enhancing the dataset’s overall utility for research and analysis. The dataset includes 21 folders, each dedicated to a specific fish species, with no additional preprocessing or normalization steps applied.

**Fig. 1:**
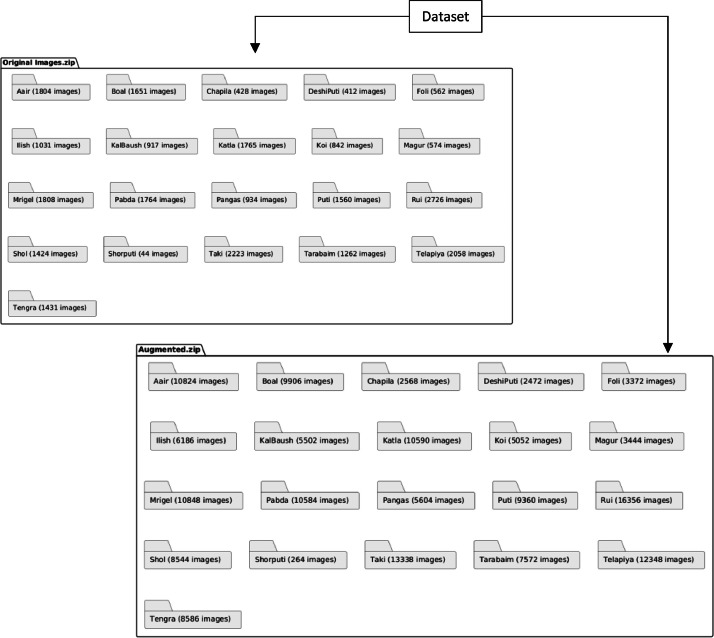
The file structure of the fish dataset.

In developing the dataset for fish species detection, the objective was to expand coverage while preserving data integrity. Images of 21 fish species were captured in local markets using high-resolution smartphone cameras, producing over 24,000 images. The compressed files—“Original.zip” (3.81 GB) and “Augmented.zip” (5.1 GB)—provide a clearly structured framework for researchers, as illustrated in Fig. 1.

## 4 Experimental Design, Materials and Methods

### 4.1 Data collection

**Fig. 2:**
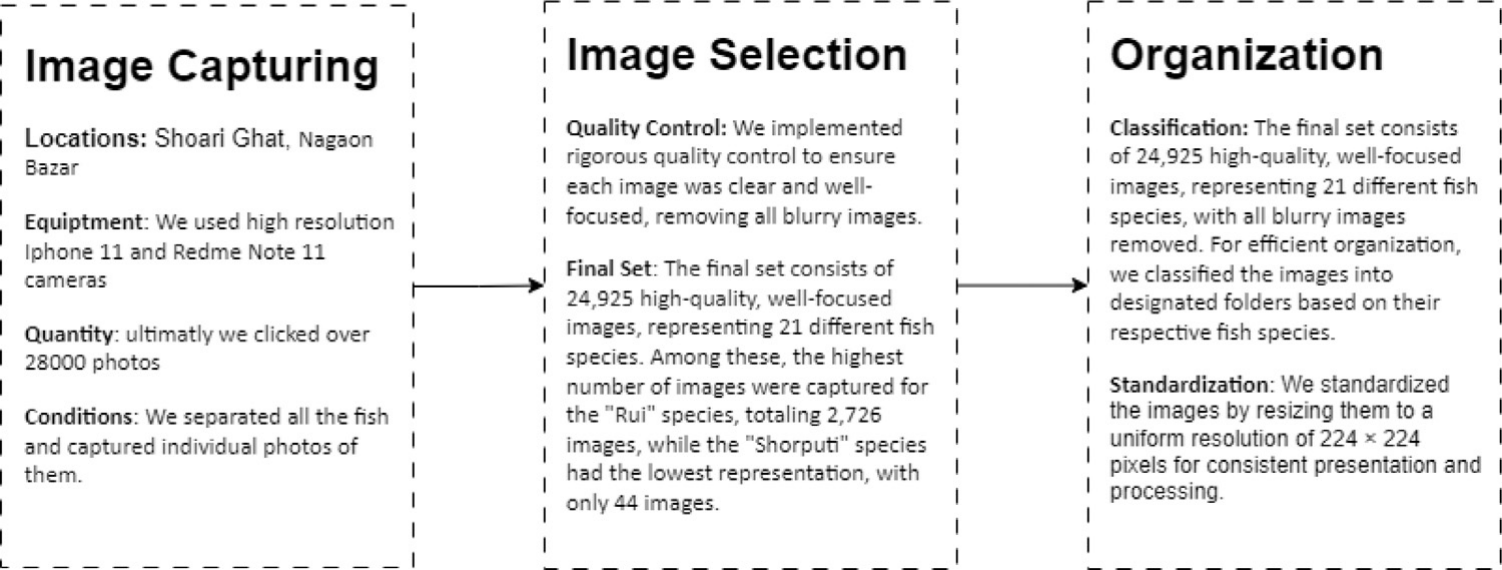
Dataset creation process.

The process began with the capture of images featuring 21 fish species at local markets, forming the foundation of the dataset. Each image was subjected to meticulous quality checks and standardization procedures to ensure consistency throughout the collection. This rigorous method was designed to provide a reliable and comprehensive resource for future research on fish species detection. Fig. 2 illustrates the overall data collection procedure.

### 4.2 Camera equipment and image acquisition

Over 24,000 images were captured using two high-resolution smartphone cameras. Each image was assessed for clarity, and those failing to meet strict quality requirements were removed during preprocessing. After this review, 24,925 high-quality images remained, each corresponding to a distinct fish species. These images were organized into separate folders based on their classification for efficient retrieval. The camera specifications are listed below:

1. **iPhone 11 Pro (Apple):**
  ○ 12 MP standard wide-angle camera (26 mm equivalent, f/1.8 aperture, OIS, focus pixels)
  ○ 12 MP ultra-wide-angle camera (13 mm equivalent, f/2.4 lens)
  ○ 12 MP telephoto camera (52 mm equivalent, f/2 lens, OIS on the Pro and Pro Max)
2. **Realme Note 11 (Xiaomi):**
  ○ 50 MP primary camera (f/1.8)
  ○ 8 MP camera (f/2.2)
  ○ 2 MP camera (f/2.4)
  ○ 2 MP camera (f/2.4)

### 4.3 Data augmentation

Random transformations were applied to each image in order to diversify the dataset and improve the robustness of subsequent machine-learning models. Initially, all original images were saved, preserving their original form for reference. Rotation [13] was then introduced by selecting a random angle between −45 and 45 degrees, while horizontal flipping [13] provided mirrored versions of the same fish. Cropping [13] was carried out by removing random margins from the edges of an image, and brightness [13] adjustments were made through factors ranging from 0.5 to 1.5. Finally, translation [13] shifted the images horizontally or vertically by a small percentage of their width or height. These transformations were systematically performed on every image, thereby creating multiple augmented versions that captured various poses, lighting conditions, and spatial arrangements of the fish species.

## Limitations

Despite the comprehensive nature of our dataset, some notable limitations may impact the model's performance:

• Single Image Limitation: The majority of the dataset comprises single photos of distinct fish species, which could make it more difficult for the algorithm to identify fish in situations with mixed species correctly. When several fish species are present in a real-world scenario, the model can have trouble accurately identifying them [10].
• Low Image Count in Some Classes: The dataset contains fewer than 500 photos for a number of species, which may impact the model's functionality. To be more precise, Chapila has 428 photographs, DeshiPuti 412 images, and Shorputi just 44 images. A lack of representation may result in decreased accuracy and generality for these underrepresented species.

## Ethics Statement

Our research adheres to ethical considerations outlined by Data in Brief, as it does not involve the use of animal or human subjects. Consequently, our dataset compilation and analysis do not raise ethical concerns related to the treatment of living beings.

## CRedit Author Statement

**Saieef Sunny**: Writing - original draft, Visualization, Validation. **Shiam Prodhan:** Conceptualization, Data curation, Writing – original draft. **Nazmuj Shakib**: Data curation, Conceptualization. **Mohammad Rifat Ahmmad Rashid:** Supervision, Visualization. **Nafees Mansoor:** Supervision, Conceptualization, Project Administration.

## Data Availability

Mendeley DataComprehensive Smartphone Image Dataset for Fish Species Identification in Bangladesh's Freshwater Ecosystems (Original data)
